# Comparison and predictors of treatment adherence and remission among patients with schizophrenia treated with paliperidone palmitate or atypical oral antipsychotics in community behavioral health organizations

**DOI:** 10.1186/s12888-017-1507-8

**Published:** 2017-10-18

**Authors:** Jeffrey P. Anderson, Zeynep Icten, Veronica Alas, Carmela Benson, Kruti Joshi

**Affiliations:** 1 GNS Healthcare, 196 Broadway, Cambridge, MA 02139-1902 USA; 2 Janssen Scientific Affairs, 1125 Trenton Harbourton Rd, Titusville, NJ 08560 USA

**Keywords:** Paliperidone palmitate, Oral antipsychotics, Schizophrenia, Adherence, Remission, Community behavioral health organization

## Abstract

**Background:**

Nonadherence to antipsychotic treatment increases the likelihood of relapse and progressive symptomatology in patients with schizophrenia. Atypical long-acting injectables, including paliperidone palmitate (PP), may increase adherence and improve symptoms. This study compared and assessed predictors of treatment patterns and symptom remission among schizophrenia patients treated with PP versus atypical oral antipsychotic therapy (OAT) in community behavioral health organizations (CBHOs).

**Methods:**

This retrospective cohort analysis evaluated 763 patients with schizophrenia and new (PP-N; *N* = 174) or continuing (PP-C; *N* = 308) users of PP, or new users of OAT (*N* = 281) at enrollment in the REACH-OUT study (2010–2013). Treatment outcomes assessed at 1 year were discontinuation, and adherence, measured by proportion of days covered (PDC) or medication possession ratio (MPR). Remission status was assessed using the Structured Clinical Interview for Symptoms of Remission (SCI-SR). A machine learning platform, Reverse Engineering and Forward Simulation (REFS™), was used to identify predictors of study outcomes. Multivariate Cox and generalized linear regressions estimated the adjusted hazard ratios (HRs) or odds ratios (ORs) with 95% confidence intervals.

**Results:**

Among PP-N users, 27% discontinued their initial treatment regimen versus 51% (*p* < 0.001) of OAT users. PP-N (vs OAT; HR = 0.49 [0.31–0.76]) users and males (HR = 0.65 [0.46–0.92]) had significantly lower rates of discontinuation. Relative to OAT, PP-N had a 36% [31%–42%] higher MPR and a 10-fold increased achievement of PDC ≥80% (OR = 10.46 [5.72–19.76]). PP users were significantly more likely to achieve remission in follow-up (PP-N vs OAT: OR = 2.65 [1.39–5.05]; PP-C vs OAT: OR = 1.83 [1.03–3.25]).

**Conclusions:**

Relative to OAT, PP was associated with improved adherence, less frequent treatment discontinuation, and improved symptom remission in this CBHO study population.

## Background

Various atypical antipsychotic medications to treat schizophrenia have been introduced to effectively manage positive symptoms, e.g., hallucinations and delusions [1]. However, nonadherence to treatment has been a common underlying issue in schizophrenia, leading to increased severity of symptoms and increased likelihood of relapse [2–4]. In addition, the increased risk of treatment nonadherence and poor health outcomes are further compounded by co-occurring tobacco, alcohol, or other substance abuse among schizophrenia patients [5]. Patients experience schizophrenia as a chronic disorder; the disease usually onsets in early adulthood and lasts throughout the patient’s lifetime [1]. Newly treated patients have an average of nine relapse episodes over 5.5 years, with the first relapse occurring at a median of 34 weeks after diagnosis [6]. As a result, schizophrenia-related costs to the healthcare system are high; a recent study has estimated that the mean cost per patient per month for patients with schizophrenia was $1387 higher ($1806 vs $419) than that for age- and gender-matched people without schizophrenia in the United States [7]. Moreover, schizophrenia patients who are nonadherent to their treatment regimen have even higher costs associated with their healthcare utilization (i.e., increased risk and duration of hospitalization) and require more emergency services [4, 8].

Atypical antipsychotic medications have been shown to be effective in managing the symptoms of schizophrenia and come in two major forms: oral formulations and long-acting injectable (LAI) formulations. LAIs work by providing stable levels of active drug within a patient that can be sustained over many weeks [9]. Since injections are administered by healthcare professionals, missed injections can easily be captured, giving the medical team the opportunity to intervene earlier [10]. LAIs have demonstrated superiority to atypical oral antipsychotic therapies (OATs) in clinical trials and observational studies [11–14]; 74% of patients in the Clinical Antipsychotic Trials of Intervention Effectiveness (CATIE) study discontinued their oral antipsychotic treatment within 18 months for various reasons [15]. Given the propensity for nonadherence in patients with schizophrenia and the consequences thereof, LAIs are a viable treatment option for schizophrenia in that they provide consistent coverage without the need for daily administration.

Paliperidone palmitate (PP) does not require oral supplementation during treatment initiation, as its pharmacokinetic profile allows both a rapid achievement of therapeutic plasma levels of paliperidone as well as a gradual and continuous release of the drug over the dosing interval, and it provides both acute symptom control and maintenance of effect [16–21]. Relative to OAT, PP has shown superiority with delayed time to relapse and improved treatment adherence [22, 23]. PP is generally well-tolerated, with the most common adverse events, occurring in less than a tenth of patients, being insomnia, worsening of schizophrenia, nasopharyngitis, headache, weight gain, and extrapyramidal symptoms [24, 25]. In a study population of previously incarcerated patients, the Paliperidone Palmitate Research in Demonstrating Effectiveness (PRIDE) trial reported a significant delay in treatment failure among those randomly assigned to PP, relative to those assigned to OAT [23]. Additionally, in a study conducted from the US healthcare payer perspective, PP was shown to be more cost-effective relative to OAT; specifically, PP patients had fewer mean annual days of relapse (8.7 days vs 17.8 days) and lower mean annual costs ($20,995 vs $22,481) [26].

Community behavioral health organizations (CBHOs) serve as the primary point of contact within the US healthcare system for patients, providing sustained outpatient care for patients who are publicly insured or uninsured and who often have chronic, severe psychiatric illnesses. These organizations offer high-quality integrated care using patient-centered approaches that focus on treatment plans to reduce and manage symptoms, as well as provide social support and services that prevent potential relapses [27]. Providing evidence of PP effectiveness in the CBHO setting is crucial given that most schizophrenia patients are treated in this setting. Additionally, much can be learned about the impact of treatment on quality of life, medication satisfaction, social functioning, symptom remission, and other patient-reported outcomes. The objectives of our study were to (1) compare the impact of PP vs OAT on medication adherence and symptom remission and (2) identify other patient factors predictive of adherence and remission among patients with schizophrenia treated with PP or OAT in CBHOs.

## Methods

### Study population

**Fig. 1:**
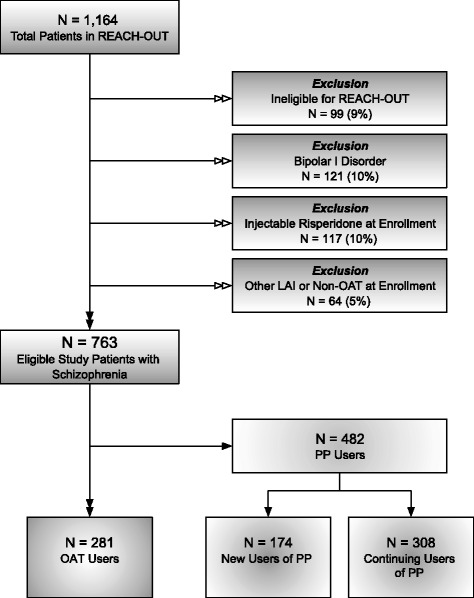
Selection of the study population. Abbreviations: LAI, long-acting injectable; OAT, oral antipsychotic therapy; PP, paliperidone palmitate; REACH-OUT, Research and Evaluation of Antipsychotic Treatment in Community Behavioral Health Organizations, Outcomes study. Notes: Reasons for REACH-OUT ineligibility (*N* = 99) included unknown or ineligible age (*N* = 34, 34%), most recent antipsychotic unknown or not study-eligible (*N* = 22, 22%), did not meet diagnostic criteria for schizophrenia or bipolar I disorder (*N* = 18, 18%), patient unwilling to complete scheduled study interviews (*N* = 17, 17%), patient participating in concurrent clinical study (*N* = 4, 4%), and patient did not consent (N = 4, 4%)

This was a retrospective cohort analysis of the prospective, observational Research and Evaluation of Antipsychotic Treatment in Community Behavioral Health Organizations, Outcomes (REACH-OUT) study [28]. This Janssen Pharmaceuticals–sponsored study of usual care of patients undergoing treatment for schizophrenia or bipolar I disorder at CBHOs was conducted between August 2010 and November 2013, approved by participating ethics committees and institutional review boards, and conducted in accordance with the ethical principles of the Declaration of Helsinki. Patients with schizophrenia or bipolar I diagnoses were enrolled in the REACH-OUT study, if they initiated treatment with risperidone: 1) 8 weeks prior to enrollment, or 2) at least 24 weeks prior to enrollment and did not have gaps between injections greater than 30 days. Patients that initiated risperidone treatment between 8 and 24 weeks prior to enrollment were excluded. Schizophrenia patients, who initiated treatment with PP or had been treated with PP any time prior to enrollment were included. Schizophrenia or bipolar I patients, who initiated treatment with an antipsychotic other than risperidone or PP within 8 weeks prior to enrollment were included. Written informed consent was obtained from all subjects prior to study enrollment. Data collection in REACH-OUT was done via patient interviews, clinician measures, and chart reviews at enrollment, and at follow-up study visits at 6 and 12 months for 1164 patients receiving care at 44 CBHOs across the United States between 2010 and 2013. For this analysis, we excluded patients found to be ineligible for REACH-OUT (*N* = 99), patients with bipolar I disorder at enrollment (*N* = 121), and patients on injectable risperidone, other LAIs, or other non-OAT antipsychotics (*N* = 181). Thus, our study population was restricted to the 763 eligible schizophrenia patients who had either initiated PP (*N* = 174) or an OAT regimen (*N* = 281) within 8 weeks of enrollment or continued previously initiated PP therapy at enrollment (*N* = 308) (Fig. 1).

The treatment status for analyses reflects the treatment regimen initiated or continued at enrollment. According to the REACH-OUT protocol, initiators of OAT were eligible if initiating an atypical oral antipsychotic medication or combination within the 8 weeks at or prior to enrollment. Similarly, new users of PP (PP-N) initiated within 8 weeks of enrollment. Continuing users (PP-C) consisted of patients initiating PP more than 8 weeks prior to enrollment. PP-C users may have initiated during or prior to the 6-month pre-enrollment study period.

### Outcomes

Treatment pattern outcomes included discontinuation, and adherence. Patients were considered to have discontinued their enrollment regimen if there was any change in treatment, including medication substitutions or stoppage. Adherence was measured using medication possession ratio (MPR), calculated for the duration of PP or OAT treatment (initiation to discontinuation), and using 1-year proportion of days covered (PDC) calculated for the duration of follow-up (1 year from enrollment). These measures were calculated using the following formulae and reported as proportions or percentages.

$$ \mathrm{MPR}=\frac{\mathrm{Days}\  \mathrm{covered}\ \mathrm{by}\ \mathrm{injection}\  \mathrm{or}\  \mathrm{prescription}\  \mathrm{from}\  \mathrm{initiation}\  \mathrm{to}\  \mathrm{discontinuation},\mathrm{n}}{\mathrm{Days}\  \mathrm{from}\  \mathrm{initiation}\  \mathrm{to}\  \mathrm{discontinuation},\mathrm{n}} $$

$$ \mathrm{PDC}=\frac{\mathrm{Days}\  \mathrm{covered}\ \mathrm{by}\ \mathrm{injection}\  \mathrm{or}\  \mathrm{prescription}\  \mathrm{from}\  \mathrm{enrollment}\  \mathrm{to}\ 12\ \mathrm{mos},\mathrm{n}}{365\ \mathrm{days}} $$

The time period for evaluation of MPR was considered to be date of initiation until discontinuation, or loss to follow-up. For PP-C users who initiated prior to REACH-OUT, MPR was calculated from first injection on study. PDC was calculated for the 365-day period from REACH-OUT enrollment to 12 months for all study patients. For patients on OAT, days covered was calculated using the days’ supply from prescription date. Maximum coverage for each PP injection was assumed to be 35 days.

Symptom severity was ascertained using a 30-item questionnaire, the Structured Clinical Interview for Symptoms of Remission (SCI-SR), administered at each study visit. The SCI-SR is a standardized definition of remission that requires a clinician’s assessment of eight Positive and Negative Syndrome Scale (PANSS) items through assigning a score between 1 (absent) and 7 (extreme) to each area: PANSS domains used in the SCI-SR definition include delusions, unusual thought content, hallucinatory behavior, conceptual disorganization, mannerism/posturing, blunted affect, passive social withdrawal, and lack of spontaneity. Remission was defined as a score of ≤3 (mild) in all eight SCI-SR symptom areas [29].

### Covariates

Several additional measures collected via patient interview and/or chart review from the REACH-OUT database were evaluated as potential covariates, including:

- Demographic information: age, gender, race, ethnicity, insurance coverage, education, and marital status
- Clinical characteristics: markers of schizophrenia disease status (duration of illness (i.e., number of years since first schizophrenia diagnosis) and disease severity), vital signs (height, weight, systolic/diastolic blood pressure), comorbid conditions (hypertension, hyperlipidemia, heart disease, diabetes, asthma, lung condition, behavioral health disorders [International Classification of Diseases codes 290–319]), and miscellaneous physical comorbidities
- Behavioral/lifestyle factors: alcohol abuse, substance use, physical activity, arrests, and smoking
- Healthcare resource utilization: hospitalization, emergency department visits, outpatient visits, and assertive community treatment (ACT)
- Patient-reported outcomes: Lehman Quality of Life (QOL) – Brief Version [30, 31]; Drug Attitude Inventory, Short Version (DAI-10) [32]; Medication Satisfaction Questionnaire [33]; Personal and Social Performance (PSP) scale [34]; and Scale to Assess Therapeutic Relationships – Patient Version (STAR-P) and Clinician Version (STAR-C) [35]

Subjective QOL domains included General Life Satisfaction, Satisfaction with Daily Activities, Satisfaction with Family Contact, Satisfaction with Social Relations, Job Satisfaction, Satisfaction with Safety, and Satisfaction with Health. These domains were scored on a scale of 1 (Terrible) to 7 (Delighted). Two objective QOL domains evaluated frequency of Family Contact and Social Contact, and were scored on a scale from 1 (Never) to 5 (≥1/day). Two additional objective QOL domains (Financial Adequacy and Victimization) evaluated the degree of financial adequacy and general victimization, ranging from 0 (No) to 1 (Yes). The DAI-10 was used to summarize patients’ attitude toward their medication regimen and was scored on a scale of −10 (poor relationship with antipsychotic medications) to +10 (positive attitude toward prescribed antipsychotics). The Medication Satisfaction Questionnaire consisted of a single evaluation of patient satisfaction with current antipsychotic medications, on a scale from 1 (Very Dissatisfied) to 7 (Very Satisfied). The PSP scale was used to measure patients’ success in psychosocial functioning, and was scored on a scale of 1 (extreme lack of autonomy in basic functioning) to 100 (excellent functioning). STAR-P and STAR-C were used to evaluate the therapeutic alliance, from the patient perspective (STAR-P) and the clinician perspective (STAR-C), respectively. These measures were scored on a scale of 0 to 48, with higher scores representing a positive patient/caregiver relationship.

### Statistical analysis

Distributions of study outcomes, by treatment status, were tabulated and evaluated using Fisher’s exact test for categorical variables or the Wilcoxon-Mann-Whitney test for continuous variables. To identify meaningful predictors of treatment outcomes, we used a machine learning analytic platform, Reverse Engineering and Forward Simulation (REFS™) [36–38]. Briefly, REFS predictive modeling generates an ensemble comprising a specified number of individual models (*N* = 50) for the outcome of interest, which is learned empirically from the data using a hypothesis-free approach based on Bayesian scoring algorithms. From the ensemble for each study outcome, predictors were ranked and evaluated by their relative selection frequency (i.e., proportion of models in the ensemble in which the variable was selected) and distributions of effect estimates, in addition to the overall predictive accuracy of the ensemble in the form of area under the (receiver operating characteristic) curve (AUC) statistics.

Subsequently, we constructed multivariable Cox (time to discontinuation) or generalized linear (all other outcomes) regression models to further evaluate effects of treatment status on study outcomes. Covariates considered for inclusion in the adjusted models were drawn based on evidence from REFS prediction model ensembles and previous literature [39]. The final adjusted models were selected using a stepwise selection algorithm based on the Akaike information criterion for each proposed model. Effect estimates are reported as beta estimates (β; linear regression), odds ratios (ORs; logistic regression), or hazard ratios (HRs; Cox regression), with 95% confidence intervals (CIs). Any reported *p* values testing statistical significance are two-sided. All statistical analyses were performed using REFS or R statistical programming software (version 3.1.1).

## Results

Demographic characteristics and other selected patient factors, by PP or OAT status at enrollment, are summarized in Table 1. The mean age for PP and OAT users was 41.1 (±12.6) years and 42.1 (±13.4) years, respectively. On average, PP users were more likely than OAT initiators to be male, single, on Medicare and/or Medicaid, smokers, diagnosed with a lung condition, and living with chronic schizophrenia for a longer duration. PP users were less likely to be Hispanic or live in a private residence (Table 1).

**Table 1:** Selected Baseline Characteristics by Treatment Status at Enrollment, REACH-OUT (2010–2013)

Variable	PP-All *N* = 482	PP-C *N* = 308	PP-N *N* = 174	OAT *N* = 281	*P* Value, PP-All vs OAT	*P* Value, PP-N vs OAT
Age (Mean ± SD)	41.12 ± 12.6	42.0 ± 12.7	39.6 ± 12.2	42.1 ± 13.4	0.440	0.069
Male	344 (71%)	219 (72%)	125 (74%)	181 (66%)	0.046	0.074
Race					0.727	0.081
White	239 (50%)	170 (57%)	69 (42%)	133 (49%)		
Black/African American	153 (32%)	80 (27%)	73 (44%)	90 (33%)		
Multiracial/other	73 (15%)	49 (16%)	24 (14%)	48 (18%)		
Ethnicity: Hispanic	65 (13%)	43 (14%)	22 (14%)	72 (26%)	<0.001	0.002
Married/committed	40 (8%)	29 (10%)	11 (7%)	35 (13%)	0.077	0.053
Medicare	238 (49%)	164 (55%)	74 (45%)	100 (38%)	<0.001	0.130
Medicaid	352 (73%)	236 (79%)	116 (70%)	169 (63%)	<0.001	0.119
Private residence	326 (68%)	213 (70%)	113 (67%)	212 (78%)	0.002	0.015
Lung condition	48 (10%)	28 (9%)	20 (12%)	15 (5%)	0.029	0.018
Smoking	346 (72%)	226 (74%)	120 (70%)	179 (64%)	0.015	0.183
Alcohol abuse	106 (22%)	73 (24%)	33 (19%)	47 (17%)	0.075	0.526
Substance abuse	114 (24%)	80 (26%)	34 (20%)	75 (27%)	0.386	0.113
Duration of schizophrenia in years (mean ± SD)	15.7 ± 12.9	16.5 ± 13.0	14.4 ± 12.5	13.9 ± 13.5	0.014	0.340
Paranoid schizophrenia	323 (67%)	203 (66%)	120 (69%)	178 (63%)	0.306	0.226
Schizophrenia severity					<0.001	<0.001
Chronic	169 (35%)	108 (36%)	61 (36%)	72 (26%)		
Subchronic	118 (24%)	68 (23%)	50 (30%)	61 (22%)		
Other/unspecified	180 (38%)	123 (41%)	57 (34%)	148 (53%)		

Notes: (1) Percentage missing: age, 2%; gender, 2%; race, 4%; Hispanic ethnicity, 3%; marital status, 2%; Medicare, 4%; Medicaid, 4%; living situation, 2%; lung condition,<1%; smoking, <1%; alcohol, 1%; substance abuse, <1%; schizophrenia duration, 4%; schizophrenia severity, 2%

(2) Percentages reported are among the nonmissing

*Abbreviations*: *OAT* oral antipsychotic therapy, *PP* paliperidone palmitate, *PP-C* continuous user of paliperidone palmitate, *PP-N* new user of paliperidone palmitate, *OAT* oral antipsychotic therapy, *SD* standard deviation

At 12 months, 308 (64%) PP users and 180 (64%) OAT users had sufficient available data to evaluate treatment discontinuation. Of these patients, 20% (*N* = 62) of PP users discontinued treatment versus 51% (*N* = 92; *p* < 0.001) of OAT users (Table 2). Additionally, PP users were significantly more adherent than OAT users to their respective treatments, by both the MPR and 1-year PDC metrics (Table 2). Specifically, mean MPR for PP users was 0.84 versus 0.52 for OAT users (*p* < 0.001); mean PDC for PP users was 0.57 versus 0.31 for OAT users (*p* < 0.001) (Table 2). When evaluating the standard threshold of ≥80% treatment coverage, the proportion adherent was significantly higher among PP users for MPR (74% vs 25%, *p* < 0.001) and PDC (44% vs 9%, *p* < 0.001) (Table 2).

**Table 2:** Treatment-Related Outcomes at 12 Months by Treatment Status at Enrollment, REACH-OUT (2010–2013)

Variable	PP-All *N* = 482	PP-C *N* = 308	PP-N *N* = 174	OAT (*N* = 281)	*P* Value, PP-All vs OAT	*P* Value, PP-N vs OAT
Discontinuation	62 (20%)	34 (17%)	28 (27%)	92 (51%)	<0.001	<0.001
MPR (mean ± SD)	0.84 ± 0.20	0.85 ± 0.17	0.82 ± 0.23	0.52 ± 0.31	<0.001	<0.001
Nonadherence (MPR <80%)	118 (26%)	74 (25%)	44 (28%)	194 (75%)	<0.001	<0.001
Adherence (MPR ≥80%)	332 (74%)	217 (75%)	115 (72%)	66 (25%)		
1-year PDC (Mean ± SD)	0.57 ± 0.39	0.60 ± 0.39	0.53 ± 0.39	0.31 ± 0.29	<0.001	<0.001
Nonadherence (PDC <80%)	270 (56%)	164 (53%)	106 (61%)	255 (91%)	<0.001	<0.001
Adherence (PDC ≥80%)	212 (44%)	144 (47%)	68 (39%)	26 (9%)		

Notes: (1) Percentages reported are among the nonmissing. Number missing discontinuation: PP-C, 33%; PP-N, 41%; OAT, 36%. Number missing MPR: PP-C, 6%; PP-N, 9%; OAT, 7%. Number missing PDC: PP-C, 0%; PP-N, 0%; OAT, 0%

*Abbreviations*: *MPR* medication possession ratio, *OAT* oral antipsychotic therapy, *PDC* proportion of days covered, *PP* paliperidone palmitate, *PP-C* continuous user of paliperidone palmitate, *PP-N* new user of paliperidone palmitate, *SD* standard deviation

**Fig. 2:**
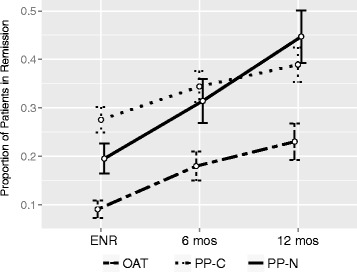
Proportion of patients in remission by study visit and treatment status, REACH-OUT (2010–2013). Abbreviations: ENR, enrollment; OAT, oral antipsychotic therapy; PP-C, continuous user of paliperidone palmitate; PP-N, new user of paliperidone palmitate

A higher proportion of PP users were in schizophrenia disease remission (all SCI-SR symptomatic domains mild or less) relative to OAT users at all time points (Fig. 2). Among those with available SCI-SR data at 12 months (PP-N, 49%; PP-C, 60%; OAT, 45%), 45% of PP-N users were in remission versus 39% of PP-C users and 23% of OAT users, respectively (*p* = 0.001). Furthermore, PP-N users in remission at 12 months represented a 25% increase from enrollment, relative to a 14% increase from enrollment among OAT initiators (Fig. 2).

Prediction model ensembles were generated for study outcomes from the REACH-OUT database using REFS. To summarize, an ensemble consisting of 50 individual models for treatment discontinuation provided moderately strong predictive accuracy (mean AUC = 0.74). Patient factors consistently selected in this ensemble as meaningful predictors of treatment discontinuation included various comorbidities, substance and alcohol abuse, hospitalizations, and poor adherence. Predictive ensembles for treatment adherence (PDC ≥80%; mean AUC = 0.76) and remission (mean AUC = 0.75) also performed well; consistent predictors included PP use and the absence of psychiatric comorbidities. Consistent, strong predictors of study outcomes identified using REFS were retained and evaluated as covariates for final generalized linear models.

In a multivariable Cox regression model, PP use was associated with a significantly lower rate of discontinuation (PP-N vs OAT: HR = 0.49, 95% CI: 0.31–0.76; PP-C vs OAT: HR = 0.28, 95% CI: 0.18–0.43). The only other statistically significant predictor of time to discontinuation in the adjusted model was gender (male vs female, HR = 0.65, 95% CI: 0.46–0.92).

PP use was significantly associated with higher MPR in an adjusted linear regression model (PP-N vs OAT, β = 0.36, 95% CI: 0.31–0.42; PP-C vs OAT, β = 0.39, 95% CI: 0.34–0.44). Additional correlates of higher MPR included shorter duration of schizophrenia (10-year increase in time from first schizophrenia diagnosis, β = −0.03, 95% CI: –0.05 to −0.01), older age (10-year increase, β = 0.03, 95% CI: 0.01–0.05), and history of arrests (β = 0.13, 95% CI: 0.01–0.25). In a multivariable logistic regression model for PDC ≥80%, initiation of PP resulted in a 10-fold increased likelihood of ≥80% treatment coverage for 1 year from enrollment, relative to OAT initiation (PP-N vs OAT, OR = 10.27, 95% CI: 5.55–19.00; PP-C vs OAT, OR = 16.47, 95% CI: 9.12–29.75). Additional patient factors associated with PDC ≥80% included ACT (OR = 3.00, 95% CI: 1.37–6.59), older age (per 10-year increase, OR = 1.29, 95% CI: 1.03–1.62), history of arrests (OR = 4.17, 95% CI: 1.14–15.32), private residence (OR = 1.89, 95% CI: 1.15–3.13), white race (vs black/African American, OR = 2.50, 95% CI: 1.49–4.17), heart disease (OR = 3.38, 95% CI: 1.36–8.72), and no history of substance abuse (OR = 2.27, 95% CI: 1.43–3.57) (Table 3).

**Table 3:** Predictors of Treatment Adherence (MPR and 1-year PDC ≥80%), REACH-OUT (2010–2013)

	MPR		PDC	
VARIABLE	β (95% CI)	*P* Value	OR (95% CI)	*P* Value
Treatment cohort:				
OAT	Reference		Reference	
PP-N	0.36 (0.31–0.42)	<0.001	10.27 (5.55–19.00)	<0.001
PP-C	0.39 (0.34–0.44)	<0.001	16.47 (9.12–29.75)	<0.001
Assertive community treatment	–	–	3.00 (1.37–6.59)	0.006
Age (10-year increase)	0.03 (0.01–0.05)	0.008	1.29 (1.03–1.62)	0.028
Arrested in last month	0.13 (0.01–0.25)	0.029	4.17 (1.14–15.32)	0.031
Private residence	–	–	1.89 (1.15–3.13)	0.012
Black/African American	–	–	0.40 (0.24–0.67)	0.001
Heart disease	–	–	3.38 (1.36–8.72)	0.010
Substance abuse	–	–	0.44 (0.28–0.70)	0.001
Duration of schizophrenia (10-year increase)	−0.03 (−0.05 to −0.01)	0.007	0.81 (0.64–1.02)	0.068

Notes: Adjusted logistic regression models, including all variables listed, plus the following additional model-specific covariates. MPR: Drug Attitude Inventory-10 score; Quality of Life social interaction domain score; hospitalizations/ED visits; insurance; gender; marital status; psychiatric comorbidities; and physical comorbidities. PDC: medication satisfaction; hospitalizations/ED visits; insurance; gender; Hispanic or other race/ethnicity; psychiatric comorbidities; physical comorbidities; alcohol abuse; and frequency of strength training

*Abbreviations*: *CI* confidence interval, *ED* emergency department, *MPR* medication possession ratio, *OAT* oral antipsychotic therapy, *PDC* proportion of days covered, *PP* paliperidone palmitate, *PP-C* continuous user of paliperidone palmitate, *PP-N* new user of paliperidone palmitate

**Fig. 3:**
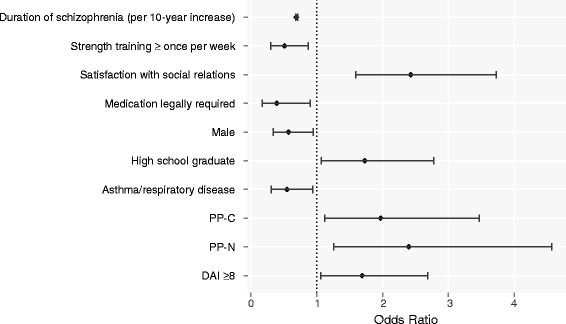
Predictors of disease remission,^a^ REACH-OUT (2010–2013). Abbreviations: DAI, Drug Attitude Inventory; PP-C, continuous user of paliperidone palmitate; PP-N, new user of paliperidone palmitate; REACH-OUT, Research and Evaluation of Antipsychotic Treatment in Community Behavioral Health Organizations, Outcomes study. ^a^Multivariable logistic mixed effects regression model for disease remission (all SCI-SR domains mild or less), adjusted for all variables shown, plus proportion of days covered (by treatment, continuous), Personal and Social Performance scale score (≥70 vs <70), general life satisfaction (QOL domain, 4–7 [mixed/satisfied] vs 1–3 [dissatisfied]), and number of outpatient visits (continuous). Odds ratios and the 95% confidence intervals included

Initiators of PP at enrollment were nearly 2.5 times as likely to achieve disease remission, relative to OAT initiators, in the adjusted model (PP-N vs OAT, OR = 2.40, 95% CI: 1.26–4.57; PP-C vs OAT, OR = 1.97, 95% CI: 1.12–3.47) (Fig. 3). Positive predictors of disease remission in the adjusted model included female gender (OR = 1.75, 95% CI: 1.05–2.94), education level (high school graduate vs less, OR = 1.73, 95% CI: 1.07–2.78), favorable attitude toward medications (DAI-10 ≥ 8 vs <8, OR = 1.69, 95% CI: 1.06–2.69), and satisfaction with social relations (QOL domain, ≥4 [mixed/satisfied] vs <4 [dissatisfied], OR = 2.43, 95% CI: 1.59–3.73). A history of asthma or respiratory disease was negatively associated with remission in this model (OR = 0.55, 95% CI: 0.31–0.94) (Fig. 3).

## Discussion

In this analysis of schizophrenia patients receiving care in CBHOs, we have compared PP and OAT use with respect to adherence and disease remission outcomes. Our results suggest that PP use is associated with increased treatment adherence, a decreased likelihood of discontinuation, and better symptom management, relative to OAT. According to our findings, predictors for discontinuation were nonadherence, OAT treatment status, healthcare resource use indicators (number of hospitalizations and ACT), substance abuse, and comorbid diagnoses. The strongest predictor of treatment adherence was PP use, which was also highly associated with remission. Other predictors of remission included female gender, education status, satisfaction with social relations, favorable attitude toward medications, and lack of lung conditions.

Poor treatment adherence needs to be addressed in order for schizophrenia patients to manage their disease and lead more satisfying lives. Our study corroborates findings from prior research demonstrating that LAIs, specifically PP, improve treatment stability among schizophrenia patients. Specifically, research in patients in Medicaid populations treated with LAIs versus OATs has shown greater antipsychotic adherence, lower rates of discontinuation (60-day continuous gap), and reduced risk of rehospitalization among those treated with LAIs versus OATs in the 6 months following a schizophrenia-related hospitalization [40, 41]. Our findings extend beyond the Medicaid population and confirm improved treatment pattern outcomes associated with PP for a heterogeneous population.

Relapse among schizophrenia patients impacts overall prognosis and healthcare utilization. Specifically, successive relapses can reduce the degree and duration of subsequent remission, worsen disability, and increase refractoriness to future treatment [42]. Furthermore, relapses are associated with high medical and nonmedical costs as well as productivity loss [43]. Therefore, managing relapses and delaying time to relapse is an important goal in schizophrenia treatment. Several studies have shown that relative to OATs, PP is associated with a significant delay in time to relapse. In a multicenter randomized trial, the Prevention of Relapse with Oral Antipsychotics versus Injectable Paliperidone Palmitate (PROSIPAL) study, 85% of PP patients were relapse-free at 469 days versus 249 days for the 85th percentile of OAT patients (*p* = 0.019) [22]. Similarly, the PRIDE study demonstrated PP superiority compared to OATs in delaying time to treatment failure during a 15-month period (PP: 39.8%; OAT: 53.7%) [23]. In addition, a higher proportion of PP users (95.2%) in PRIDE were adherent (MPR ≥80%) relative to OAT users (77.2%) [23]. Our findings confirm improved treatment adherence outcomes and extend beyond the PRIDE population of previously incarcerated patients.

Finally, in a claims analysis by Marcus et al., PP was significantly protective against nonadherence (PDC <80%, OR = 0.34, 95% CI: 0.22–0.54), discontinuation (≥60-day gap, OR = 0.39, 95% CI: 0.23–0.66), and rehospitalization (OR = 0.53, 95% CI: 0.30–0.94), as compared to OATs [44]. Our research complements these existing findings in suggesting the superiority of PP over OATs with respect to remission and treatment outcomes in a CBHO population.

Our use of REFS was advantageous in that it identified relevant predictors of study outcomes with more resolution and rigor than other available variable selection methods. REFS generates an ensemble (series) of prediction models, based on Bayesian mathematics, as opposed to a single maximum likelihood model, which can be susceptible to misspecification or chance (particularly in smaller data sets). From the distribution of effect estimates in the resulting ensembles, the degree of uncertainty of an effect estimate could be more closely assessed. Given the unique nature of the data collected in REACH-OUT, we believe this empirical, data-driven approach suitably complemented previous literature in specifying the final models presented here, with the goal of optimizing predictive value.

There are some limitations that should be considered when interpreting the results of our study. First, statistical power may have been suboptimal due to a relatively small sample size (particularly for PP initiators) and a limited follow-up period. However, we were able to detect several statistically significant differences in outcomes between the study cohorts. Second, the cohort of PP users was a combination of PP-N and PP-C users. To address this discrepancy, we adjusted for new and continuing PP status in primary models. OAT users were exclusively initiators at enrollment. Due to the design of the chart review form, these patients could not be further stratified by type of OAT, which may highlight additional treatment differences associated with the study outcomes. Third, calculations of adherence were inherently inconsistent due to the nature of LAI vs oral antipsychotic medications. Specifically, we have assumed PP users were covered for 35 days, where treatment coverage for OAT users was determined by days of supply. However, days’ supply may overestimate adherence among OAT users, since their actual fulfillment of the treatment was not captured. Finally, PDC was calculated for all eligible participants at enrollment, regardless of actual follow-up status, which may or may not have been differential by treatment status. To validate PDC calculations, we have also estimated MPR, which was applied only while patients were specifically under observation.

In this study of schizophrenia patients receiving care at CBHOs, our results suggest that PP users benefit from treatment stability as well as improved clinical management of symptoms. These findings were generally confirmed in our comparisons of initiators of PP versus initiators of OAT, suggesting potential clinical utility for PP beyond salvage therapy for patients who fail on OAT regimens. All analyses are exploratory and to be interpreted with caution. Further studies of the impact of LAI use on clinical outcomes of schizophrenia in alternative study populations are warranted.

## Conclusions

Long-acting injectable PP was associated with improved adherence and lower treatment discontinuation. Additionally, PP users demonstrated significant association with achievement of disease remission relative to OAT users in this study of adults with schizophrenia receiving care at CBHOs.

## Abbreviations

ACT: assertive community treatment
AUC: area under the curve
CATIE: Clinical Antipsychotic Trials of Intervention Effectiveness study
CBHO: Community Behavioral Health Organization
CI: confidence interval
DAI: Drug Attitude Inventory
HR: hazard ratio
LAI: long-acting injectable
MPR: medication possession ratio
OAT: oral antipsychotic therapy
OR: odds ratio
PANSS: Positive and Negative Syndrome Scale
PDC: proportion of days covered
PP: paliperidone palmitate
PP-C: continuous user of paliperidone palmitate
PP-N: new user of paliperidone palmitate
PRIDE: Paliperidone Palmitate Research in Demonstrating Effectiveness study
PROSIPAL: Prevention of Relapse with Oral Antipsychotics versus Injectable Paliperidone Palmitate
PSP: Personal and Social Performance scale
REACH-OUT: Research and Evaluation of Antipsychotic Treatment in Community Behavioral Health Organizations Outcomes study
REFS™: Reverse Engineering and Forward Simulation
SCI-SR: Structured Clinical Interview for Symptoms of Remission
STAR-C: Scale to Assess Therapeutic Relationships – Clinician Version
STAR-P: Scale to Assess Therapeutic Relationships – Patient Version
